# Discovery of Antimicrobial Agents Based on Structural and Functional Study of the *Klebsiella pneumoniae* MazEF Toxin–Antitoxin System

**DOI:** 10.3390/antibiotics13050398

**Published:** 2024-04-26

**Authors:** Chenglong Jin, Sung-Min Kang, Do-Hee Kim, Yuno Lee, Bong-Jin Lee

**Affiliations:** 1 The Research Institute of Pharmaceutical Sciences, College of Pharmacy, Seoul National University, Seoul 08826, Republic of Korea; senglyong@snu.ac.kr; 2 Mastermeditech Ltd., Gangseo-gu, Seoul 16499, Republic of Korea; 3 College of Pharmacy, Duksung Women’s University, Seoul 01369, Republic of Korea; smkang@duksung.ac.kr; 4 College of Pharmacy, Sookmyung Women’s University, Seoul 04310, Republic of Korea; dohee.kim@sookmyung.ac.kr; 5 Korea Research Institute of Chemical Technology, Korea Chemical Bank Daejeon, Daejeon 34114, Republic of Korea; yunolee1@krict.re.kr; 6 College of Pharmacy, Ajou University, Yeongtong-gu, Suwon 16499, Republic of Korea

**Keywords:** *Klebsiella pneumoniae*, toxin–antitoxin system, MazEF

## Abstract

*Klebsiella pneumoniae* causes severe human diseases, but its resistance to current antibiotics is increasing. Therefore, new antibiotics to eradicate *K. pneumoniae* are urgently needed. Bacterial toxin–antitoxin (TA) systems are strongly correlated with physiological processes in pathogenic bacteria, such as growth arrest, survival, and apoptosis. By using structural information, we could design the peptides and small-molecule compounds that can disrupt the binding between *K. pneumoniae* MazE and MazF, which release free MazF toxin. Because the MazEF system is closely implicated in programmed cell death, artificial activation of MazF can promote cell death of *K. pneumoniae*. The effectiveness of a discovered small-molecule compound in bacterial cell killing was confirmed through flow cytometry analysis. Our findings can contribute to understanding the bacterial MazEF TA system and developing antimicrobial agents for treating drug-resistant *K. pneumoniae*.

## 1. Introduction

*Klebsiella pneumoniae* is a Gram-negative, encapsulating, and nonmotile bacterium in the *Enterobacteriaceae* family [1]. The clinical diseases caused by *K. pneumoniae* include pneumonia, thrombophlebitis, urinary tract infection, cholecystitis, diarrhea, upper respiratory tract infection, wound infection, osteomyelitis, meningitis, bacteremia, and sepsis [2,3]. Once infection with *K. pneumoniae* occurs, antibiotic treatment should be applied. Current therapies for *K. pneumoniae* depend on a two-week treatment with third or fourth generation cephalosporin or a quinolone as monotherapy. In some cases of severe infections, aminoglycoside or carbapenem is also used [4,5]. However, substantial evidence of antibiotic resistance in *K. pneumoniae* has accumulated worldwide. For example, *K. pneumoniae* producing extended beta-lactamase can hydrolyze and incapacitate cephalosporins. Furthermore, there are extremely drug-resistant strains of *K. pneumoniae* that are even resistant to carbapenems. Therefore, the development of new antibiotics to eradicate *K. pneumoniae* by exploiting new therapeutic strategies is urgently needed [6,7].

Initially, it was reported that only bacterial cells inheriting the toxin–antitoxin (TA) operon from their parent cells could survive normally. Thus, TA systems were initially recognized to be involved in the maintenance of plasmids in bacteria [8]. Subsequently, it was revealed that TA systems are more broadly involved in numerous physiological processes of bacteria, such as gene regulation, cell growth, survival, and apoptosis [9]. TA systems consist of operons encoding toxin and antitoxin genes adjacently, which are transferred genetically to daughter cells [10]. Typically, the transcription of the antitoxin gene occurs simultaneously with the toxin gene, and the antitoxin protein neutralizes the toxic effect of the toxin protein [11]. However, in unfavorable environments for bacteria, such as abnormal temperatures, nutrient deficiencies, bacteriophage infections, and antibiotic treatments, antitoxins degrade and disappear, resulting in an increased level of free toxins that trigger cell growth arrest or bacterial cell death [12,13].

TA systems can be mainly classified into six categories based on the nature and neutralization mechanisms of antitoxins in each type. In type I TA systems, the antitoxin exists in the form of antisense RNA, which disrupts toxin mRNA, thereby inhibiting toxin synthesis [14]. In type II TA systems, antitoxin is a small protein. This protein antitoxin acts as an inhibitor binding to protein toxin by forming protein complex [15,16]. In type III TA systems, antitoxins take the form of RNA and form an RNA-protein complex with its coupled toxin protein [17]. In type IV TA systems, although toxin and antitoxin do not interact directly, they act as opposing factors on the same protein, such as the cases of FtsZ and MreB [18]. Additionally, antitoxins in type V TA systems act as ribonuclease and degrade their cognate mRNA toxins [19]. Antitoxins in type VI systems translocate their cognate toxins to cellular protease and promote the degradation of toxin [20].

In the *K. pneumoniae* MGH 78,578 strain, there are pairs of type II TA systems. Among those TA systems, two systems (HipAB and MazEF systems) are reported as RelBE systems and the others remain as hypothetical [21]. Generally, antitoxins in type II TA systems have a labile nature and are susceptible to cellular proteases. Because toxins are thermodynamically stable, degradation or artificial removal of antitoxin can generate the release of free toxin from the TA protein complex, inducing growth inhibition or the death of bacterial cells [22]. Especially, the MazEF system is related to programmed cell death and freely released MazF toxin can also result in bacterial cell death [23,24]. Thus, it can be discussed that regulating the level of free MazF toxin might be utilized as a strategy to promote bacterial cell death [25].

Here, we present the putative antimicrobial agents discovered by using the structure of the TA system protein. Over a decade ago, there was proof that peptide derivatives could disrupt the TA complex [26]. Also, direct experimental demonstration of the disruption of *Mycobacterial tuberculosis* VapBC TA complex was reported [27]. This research reinforces the principle of the ‘artificial activation of toxin’ through the *K. pneumoniae* MazEF system. Structural analysis of the binding interface between MazE and MazF made it possible to design peptides mimicking the binding region to artificially disrupt the MazEF complex. Artificial activation of MazF was induced by those peptides, and their efficacies were verified in vitro as well as in vivo. Freely released MazF resulting from complex disruption was confirmed by an in vitro ribonuclease activity assay. The effect of designed peptides on cell viability was also confirmed by an antimicrobial activity test. Additionally, through molecular docking simulation, small molecules capable of blocking the binding pocket between MazE and MazF were designed, and the final candidate compounds were selected in an experimental manner similar to those of peptides. Our findings will contribute to the understanding of the bacterial MazEF TA system and to the development of antimicrobial agents for the treatment of drug-resistant *K. pneumoniae* infection.

## 2. Results and Discussion

### 2.1. Summary of MazEF Complex Structure

The information of genetic constructs, expression/purification of the proteins, crystallization conditions, data collection, data processing, and refinement statistics regarding the deposited structure (PDB ID: 7BYE) has been reported in a previous paper [28].

In the subunit of MazEF, the MazE monomer was positioned between symmetrically arrayed MazF dimers [28]. The MazE monomer interacts with the MazF homodimer via its long C-terminus loop, which wraps around the MazF dimer and extends further toward the empty edge between MazF monomers. The MazE antitoxin interacts with the MazF toxin via two binding modes, namely, hydrophobic and hydrophilic interactions. The long C-terminus loop of MazE contributes to binding with MazF through hydrophobic interactions involving the following MazE residues: L50, L53, L54, Y66, L67, and M75 (chain A) and the following MazF residues: L14, F16, A30, V47, P49, P58, P59, L74, L81, M105, and I109 (chain B) and F16, L37, F38, and V41 (chain C) [28].

Additionally, the C-terminus of MazE contributes to the hydrophilic interaction between MazE and MazF with several residues involved in hydrogen bonding or salt bridges. In detail, the following residues are necessary for forming hydrogen bonds: E65, Y66, D69, S70, K73, E74, and M75 of MazE (chain A); N17, P18, G43, T51, Q77, K79, D82 and R86 of MazF (chain B); and N17, K79, D82 and R86 of MazF (chain C) [28]. In addition, 13 salt bridges are formed between MazE (chain A) and MazF (chain B) and three salt bridges are formed between MazE (chain A) and MazF (chain C).

**Figure 1:**
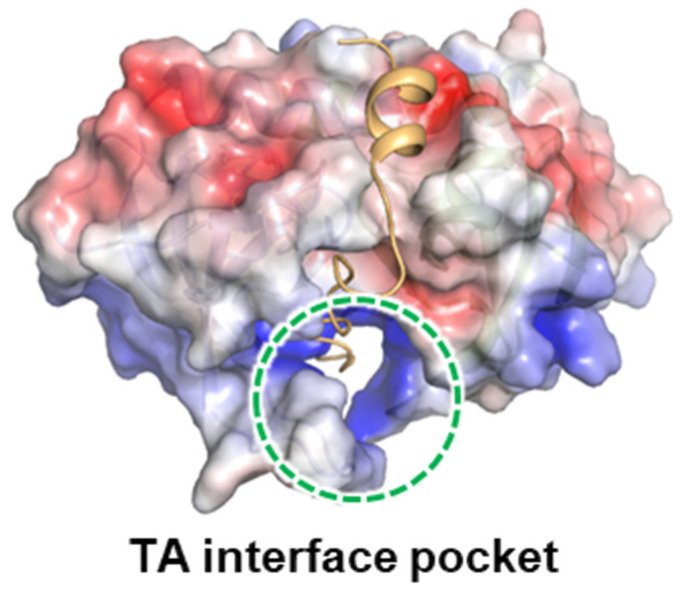
Structural summary of MazEF. Electronic surface potential of MazEF. TA interface pocket is denoted.

In the surface of MazEF, the interactions between MazF monomers occur especially in the ‘TA interface pocket’ [29,30] (Figure 1). Notably, in the *K. pneumoniae* MazEF TA interface pocket, the major MazE-binding region is highly positively charged.

### 2.2. Molecular Docking of Small-Molecule Inhibitors Interfering with the Formation of the MazEF Complex into the Surface Binding Pocket

**Figure 2:**
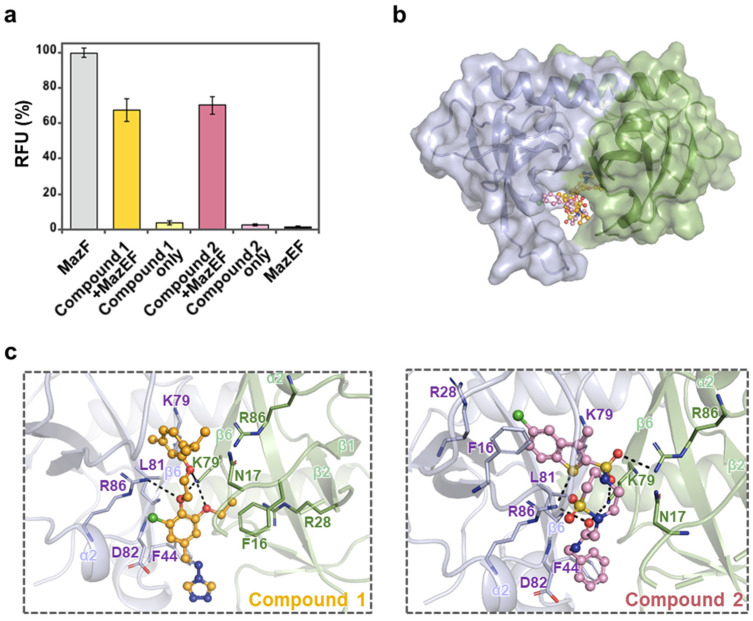
Two compounds were found to interfere with the formation of the MazEF complex and release MazF toxin. (**a**) Statistical representation of fluorescence measurements using Compound **1** and Compound **2**. The RFU obtained with 4 μM MazF monomer was taken as 100%, and that obtained with 4 μM MazEF was regarded as 0% (Materials and Method Section 3.1). (**b**) The surface of MazF with ball and stick representations of two compounds. Compound **1** and Compound **2** ‘attack’ the TA interface pocket and disrupt the binding interface. Consequently, free MazF toxins bearing active sites are released. (**c**) Binding modes of Compound **1** and Compound **2** obtained from the molecular modeling study. In the cartoon diagram, Compounds **1** and **2** are shown in yellow and pink, respectively. Ligand-binding residues of MazF are shown in ball and stick representation and labeled. The residues involved in hydrogen bonds are represented by black dashes.

Detailed structural information of the *K. pneumoniae* MazEF complex revealed the binding interface between the MazE antitoxin and MazF toxin. Based on the toxin–antitoxin binding pocket, we selected 400 putative inhibitors from the Korea Chemical Bank (KCB) by a structure-based virtual screening approach. The majority of these compounds are distributed in an MW range of 300−500 Da with an average of ~5.1 HB acceptors, ~2.2 HB donors, and 6.8 rotatable bonds. The predicted AlogP values for most of the compounds are in the range of 0.5−5, with an average value of 2.8. Initially, 2 nM of synthetic RNA substrates, 4 μM concentrations of the MazEF complex, and 5 μM small-molecule compounds were employed in the initial fluorescence assay execution. Subsequently, observations were made to determine the extent to which the MazEF complex was effectively disrupted based on the outcomes. After initial screening, two compounds (Compounds **1** and **2**) were found to disrupt the MazEF complex and release approximately 70% of free MazF toxin (Figure 2a). The compounds themselves were also assayed as controls. The results showed that Compounds **1** and **2** themselves did not exhibit ribonuclease activities. Thus, it can be confirmed that these two compounds indeed bind to the TA interface pockets and disrupt the binding interface.

As mentioned above, the MazE monomer interacts with the MazF homodimer via its long C-terminal loop in the normal state. Interestingly, most of the interactions between MazE and MazF are located in the TA interface pocket. Thus, if a specific compound displays high affinity for the TA interface pocket of MazF, free MazF toxin will be produced. This suggests that if a specific compound displays high affinity for the TA interface pocket of MazF, free MazF toxin will be produced due to the interactions between MazE and MazF being located in the TA interface pocket.

To better understand how compounds competitively bind with MazF toxin, the resulting docking poses of compounds (Compounds **1** and **2**) obtained from the molecular docking simulation performed by CDOCKER implemented in Discovery Studio were analyzed and compared with the crystal structure of *K. pneumoniae* MazEF. The docking models of the two compounds bound to the MazF dimer showed the predicted binding modes of these compounds. Both compounds competitively bind with MazF at TA interface pockets (Figure 2b,c). In detail, Compound **1** formed strong hydrogen bonds with K79 of one MazF monomer (chain C) and R86 of another MazF monomer (chain B). Additionally, residues F44, K79, D82, and R86 from chain B and N17, R28, and R86 from chain C formed van der Waals interactions with Compound **1**. Residues K79, L81, and R86 from chain B and F16 from chain C formed alkyl interactions and π-alkyl interactions, respectively. Similarly to the hydrogen bonds of Compound **1**, Compound **2** was predicted to form strong hydrogen bonds with K79 and R86 from chain C and R86 from chain B. Furthermore, residues F44, L81, and D82 from chain B and N17 from chain C formed van der Waals interactions with Compound **2**. In this case, the F16 from chain B formed strong π-π stacking and a π-alkyl interactions with the chlorobenzothiophene group of the compound. Residues R28 and K79 from chain B have additional alkyl interactions with the chlorobenzothiophene moiety.

These assorted interactions between the inhibitor compounds and the MazF dimer replaced the previous interactions between the MazF dimer and MazE. In particular, K79, R86, and F16 were the most important residues in this binding.

### 2.3. Design of Antimicrobial Peptides Mimicking the Binding Interface between MazE and MazF

**Figure 3:**
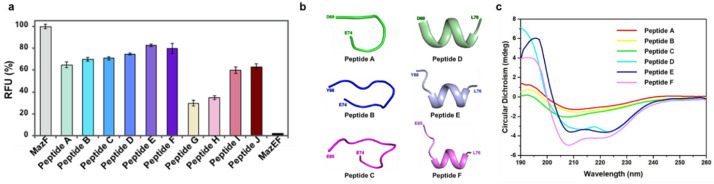
Efficacy of complex disruption using the designed peptides. (**a**) Peptides A−F represent MazE-mimicking peptides, while peptides G–J represent MazF-mimicking peptides. The RFU obtained with 4 μM MazF monomer was taken as 100%, and that obtained with 4 μM MazEF was regarded as ~0%. Error bars represent the standard error of the means from three independent experiments. (**b**) Prediction of the designed peptide by PEP-FOLD4. Interestingly, when M75 and L76 were added to existing peptides, their secondary structures changed from random coil to α-helix. (**c**) Validation of the secondary structure of the designed peptides using circular dichroism (CD).

After finding two compounds that can disrupt the MazEF complex, we made further efforts to design several peptides that play roles similar to that of small-molecule inhibitors (Table 1). Theoretically, these mimicking peptides should compete with MazE or MazF and hinder the formation of the MazEF complex. If a designed peptide displays high affinity for the corresponding binding region, free MazF will be released, resulting in increased RNase activity [31]. All 10 designed peptides exhibited various RNase activities in the in vitro assay. Among them, MazE-mimicking peptides (Peptides A–F) disrupted the interaction of the MazEF complex by up to 80% (Figure 3a), while only approximately 60% of the MazEF complex was disrupted by using MazF-mimicking peptides (Peptides G–J) (Figure 3a). As mentioned above, the MazE antitoxin interacts with the MazF toxin via three types of noncovalent interactions: hydrogen bonds, salt bridges, and hydrophobic interactions. Based on the tertiary structure of the MazEF complex, residues involved in the binding interface were found using PISA [32] and the PIC server [33]. As a result, among the residues of MazE, D69 is a key residue that possesses the largest number of interactions in the binding interface. E74, K73, Y66, and E65 are also involved in the interactions in descending order. Interestingly, M75 and L76, which are the last two residues in the tail of MazE, are involved only in the interactions with chain C of MazF. Furthermore, among the residues of MazF, R86 is a key residue in the binding interface, which possesses more than 15 interactions with MazE. In addition, Q77, K79, and D82 are also involved in the binding interface. Therefore, it can be inferred from the structural interactions (Figure 1) that the RNase activity of the designed peptides correlates with their physiochemical properties. For MazE-mimicking peptides, the main framework of the peptide is DSQGKE (Peptide A), which contains the crucial residues (underlined) involved in the binding interface. This peptide disrupted approximately 65% of the MazEF complex, while YLCDSQGKE (Peptide B) and EYLCDSQGKE (Peptide C) did not exhibit significant differences from Peptide A (Figure 3a). In contrast, there was a 10−15% increase in RNase activity when M75 and L76 were added to the previous peptides. Among them, YLCDSQGKEML (Peptide E) and EYLCDSQGKEML (Peptide F) mostly disrupt the MazEF complex by up to 80−83%. Furthermore, the secondary structures of the designed peptides were predicted using PEP-FOLD [34], which aims to achieve the de novo modeling of secondary conformations for peptides in aqueous solution. Interestingly, M75 and L76 affect not only the functions of the peptides but also their conformations. It can be predicted that peptides A−C lacking M75 and L76 exist as random coil, while peptides D−F, including M75 and L76, possess secondary structures containing α-helices (Figure 3b). The prediction is highly consistent with the results of circular dichroism (CD) (Figure 3c). For MazF-mimicking peptides, although there was no significant difference between KSLD (Peptide G) and QVKSLD (Peptide H), a dramatic increase in RNase activity was detected using KSLDWKAR (Peptide I) and QVKSLDWKAR (Peptide J). This illustrates that R86 is indispensable for competing with MazF toxin, while Q77 is not essential for MazF-mimicking peptides to disrupt the TA complex.

Based on the in vitro RNase assay, we found several peptides that can liberate MazF toxin from the MazEF complex by competitively occupying the binding interface. Among them, YLCDSQGKEML (Peptide E) and EYLCDSQGKEML (Peptide F) were chosen to conduct the following in vivo cell viability assay, since they had a relatively large effect on RNase activity compared to other peptides in vitro. However, the designed peptides could be made to be more effective by modifying several factors that affect the peptide activities. For example, modified peptides with better folding and higher α-helical content exhibited greater antimicrobial activity [35]. Thus, peptide modification using conjugation strategies and surface modulation could be conducted to obtain better structure folding and permeability. However, it should be noted that α-helical peptides could exhibit hemolytic activities in some cases [36]. It is suggested that the helicity of designed peptides could be adjusted using D-amino acids. This can decrease hemolytic activity and enhance stability against proteolytic cleavage. Furthermore, it must be considered that the hydrophobicity of peptides could affect their permeability. A previous study showed that the hydrophobicity of designed peptides not only affected their activity but also changed the range of targets. For instance, there is an optimal window of hydrophobicity beyond which the activity of the designed peptide decreases rapidly [37]. Additionally, some synthetic analogs of peptides, such as magainin, can kill both Gram-negative and Gram-positive bacteria when they are modified to have higher hydrophobicity [38]. Last, the cytotoxicity of the designed peptide would be affected by changing the amino acid content. It was reported that P60.4 used against methicillin-resistant *Staphylococcus aureus* (MRSA) exhibited lower cytotoxicity when it was modified by replacing neutral amino acids (Asn and Gln) with positively charged residues (Arg) [39].

### 2.4. Confirmation of the Possibility of Cell Killing by Designed Inhibitors

Based on the results of the in vitro fluorescence quenching assay, several significant hits (both peptides and small molecules) were found. Clearly, our ultimate objective is to identify candidates capable of rapidly killing bacteria, consequently inducing cell death. To evaluate the antimicrobial activity of the hits mentioned above, minimum antimicrobial concentration (MIC) values were measured using *K. pneumoniae* ATCC 700721. All of the inhibitors exhibited detectable activities against *K. pneumoniae* cell lines. Among them, Compound **1** had the smallest MIC value of 63 μM, while that of Compound **2** was much higher than the former. The two peptides exhibited the same MIC value of 125 μM.

In vivo experiments found several antimicrobial agents, although their MIC values are relatively high at present. As mentioned above, current therapies for *K. pneumoniae* depend on cephalosporins or quinolones. Their mechanisms of action (MOAs) are described next. Cephalosporins mimicking D-Ala-D-Ala irreversibly binds to penicillin-binding proteins (PBPs) to disrupt the synthesis of the peptidoglycan layer forming the bacterial cell walls [40]. In contrast, quinolones for many Gram-negative bacteria inhibit bacterial DNA gyrase, thereby inhibiting DNA replication and transcription [41]. Notably, our antimicrobial agents designed by structure-based information use a completely new strategy to ‘attack’ *K. pneumoniae* bacteria. We exploited toxin–antitoxin (TA) systems existing in bacteria, which respond to stresses and induce programmed cell death [42]. In particular, the concept of ‘conditional cooperativity’ in TA systems was applied [43]. In a growing bacterial population, antitoxin alone or antitoxin–toxin complex specifically bind to their own promotors, resulting in moderate repression and robust repression, respectively. In contrast, exposure to stress, such as antibiotics or nutrient deprivation, leads to the activation of bacterial proteases, especially Lon and ClpXP, that preferentially cleave the antitoxin, resulting in a transient excess of toxin [43,44,45]. After that, the additional toxin interacts with the existing complex, causing conformational changes in the complex and bringing about robust transcription. In this autoregulatory mechanism, the former is regarded as negative feedback, while the latter is regarded as positive feedback [45].

Most TA systems examined in previous studies exhibit conditional cooperativity, with exceptions such as the DinJ/YafQ and MqsRA systems [43]. Thus, our antimicrobial agents were speculated to function as follows. When our compounds or peptides penetrate *K. pneumoniae* cells, they primarily disrupt the existing MazEF complex, resulting in an excess of active toxin. These active toxins break the balance between MazE antitoxins and MazF toxins, greatly increasing the ratio of toxin to antitoxin. Then, positive feedback loops are induced, leading to overexpression of the MazEF complex. Thus, our antimicrobial agents disrupt the MazEF complex and release free toxins, which results in rapid killing of the target bacteria. It has of course been suggested that the amounts of toxin alone, antitoxin alone, and toxin–antitoxin complex should be detected in real time. However, measuring toxin and antitoxin levels in single cells in vivo is a challenging task with two main obstacles [45]: tagging with fluorescent reporters would decrease the stability of the complexes, and tagging toxins with fluorescent probes could interfere with the toxin activities, resulting in experimental errors [46].

## 3. Materials and Methods

### 3.1. In Vitro Ribonuclease Assay

To validate the ribonuclease activity of MazF toxin, an RNase Alert Kit (IDT, Coralville, IA, USA) was used. The principle is as follows. In the normal state, a fluorophore is covalently attached to one end of a synthetic RNA strand and is quenched by a quencher group at the other end of the RNA strand. However, the synthetic RNA is digested and the quencher is released when the ribonuclease interacts with substrates. Then, the released fluorophore emits fluorescence at 520 nm upon excitation at 490 nm. The resulting relative fluorescence units (RFU) were detected using a SPECTRAmax GEMINI XS spectrofluorometer (Molecular Devices, San Jose, CA, USA). All experiments were performed in triplicate.

### 3.2. In Vitro Assays of Complex Disruption by Small Molecules

Based on the tertiary crystal structure of the MazEF complex, we tried to discover small-molecule compounds, which can ‘attack’ the binding pocket between MazE and the MazF complex to disrupt the MazEF complex. Based on the tertiary structure of MazEF, 400 different candidates were chosen from Korea Chemical Bank, which has 610,000 different kinds of compounds in their library (http://www.chembank.org, accessed on 21 April 2024). Throughout the virtual screening, a total of 400 small-molecule candidates capable of binding to the pocket of the MazF surface were selected. By using these compounds, in vitro assay was conducted. Theoretically, when the inhibitor compounds are dissolved in the buffer containing the MazEF complex, they should compete with the corresponding antitoxin protein for binding to MazF toxin. Then, if the compounds are successfully bound, the free toxin will be released from the complex and act as a ribonuclease [31]. The process was monitored by a fluorescence assay, which was mentioned above. In each test, 2 nM of synthetic RNA substrates were used. The concentrations of the MazEF complex and small-molecule compounds were fixed at 4 μM and 5 μM, respectively. The final buffer prepared in this assay was 20 mM Tris, pH 8.0, and 200 mM NaCl. Small-molecule compounds and synthetic RNA substrates were lyophilized and diluted using the same final buffer. The MazEF complex was incubated with the compounds and synthetic RNA substrates for 30 min at 37 °C before fluorescence measurement.

### 3.3. Molecular Docking Simulations

To screen the primary hit compounds, molecular docking simulation was performed with 7155 diverse set compounds representing a total of ~610,000 KCB compounds. The target protein is the MazF homodimer (PDB ID: 7BYE), which is in the state of complex with the MazE monomer. The binding site setting with a sphere of 14.3 Å radius was defined from the structure of the C-terminal tail of the MazE monomer. The 3D structure of the missing loop of the MazF homodimer was built by the Build homology model protocol implemented in Discovery Studio (DS) 2018 [47]. The CHARMm-based docking tool, CDOCKER [48], equipped in the DS was used to perform the docking simulation. The CDOCKER has an advantage for the enhanced flexibility of ligand conformation refined by random rotation and grid-based simulated annealing through high temperature. The resulting hit compounds were ranked by the negative CDOCKER interaction energy (including van der Waals and electrostatics) and were further analyzed by 2D diagram visualization and monitor command in DS 2018 software.

### 3.4. In Vitro Assays of Complex Disruption by Peptides

In addition to the disruption assay using small molecules, 10 short peptides that mimic the binding region between MazE and MazF were designed based on the tertiary crystal structure of the MazEF complex and purchased from ANYGEN (http://www.anygen.com, accessed on 21 April 2024) (Table 1). Among the peptides tested, six were designed to mimic the region on MazE where it binds to MazF, while the remaining four were designed to mimic the region on MazF where it binds to MazE. This design was chosen to investigate the interactions between MazE and MazF and their respective binding regions. The main principle and process were consistent with those of complex disruption by small molecules, which has been mentioned above. The concentration of the MazEF complex was fixed at 4 μM, and the concentration of competing peptides was 20 μM. Furthermore, all of the proteins and peptides used in this assay were prepared in 20 mM Tris, pH 8.0, and 200 mM NaCl. The MazEF complex was incubated with the peptides for 30 min at 37 °C before fluorescence measurement.

### 3.5. Antimicrobial Activity Test

The antimicrobial activities of peptides and small-molecule hits were evaluated by measuring their minimum inhibitory concentration (MIC) values using the serial dilution method. The bacterial strain used in this assay was *K. pneumoniae* ATCC 700721. The cells were grown for 24 h at 37 °C in the presence of various concentrations of peptides or small molecules (0.49 μM−500 μM). The MIC was defined as the lowest concentration that can completely inhibit the growth of bacteria. Each test was conducted in duplicate.

## 4. Conclusions

The information about the crystal structure of *K. pneumoniae* MazEF complex made it possible to design several peptides or small molecules to artificially disrupt the MazEF complex. Small molecules triggering the TA complex to release free toxin have been previously reported in the *Klebsiella pneumoniae* VapBC system [49]. Our study is the report about small molecules disrupting the TA complex of *Klebsiella pneumoniae* MazEF. In our study, antimicrobial agents could be found although their MIC values are relatively high at present. Further effort to address these drawbacks can be made by exploring the potential use of the identified compounds as adjuvants with existing therapies, along with strategies such as altering the application of antimicrobial agents and modifying the compounds or peptides themselves. This strategy poses a greater challenge, as it involves combining cephalosporins with our agents. Antibiotics are known to send stress signals to bacteria harboring TA systems, including the MazEF system. Thus, low concentrations of cephalosporins may trigger a positive feedback loop in the TA systems of target bacteria, making lower concentrations of our agents sufficient to inhibit or kill the cells. However, controlling the concentration of cephalosporins and the timing of applying our agents to bacteria may prove difficult. The methods of peptide modification were introduced in detail in the previous subsection. For small molecules, several factors, such as polarity, molecular weight, amine, amphiphilic and rigid, and low globularity, would be computationally analyzed and modified to promote the rapid traversal of the membranes of Gram-negative bacteria [50]. Furthermore, new derivatives could be designed on existing scaffolds with more interactive groups to be more effective in disrupting the MazEF complex. In conclusion, with further research, our antimicrobial agents could have the potential to act as antimicrobial candidates to treat drug-resistant *K. pneumoniae*.

## Figures and Tables

**Table 1:** Peptides used to disrupt the binding interface of MazEF.

Name	Mimicked Protein	Residues (Start–End)
Peptide A	MazE	DSQGKE (69–74)
Peptide B	MazE	YLCDSQGKE (66–74)
Peptide C	MazE	EYLCDSQGKE (65–74)
Peptide D	MazE	DSQGKEML (69–76)
Peptide E	MazE	YLCDSQGKEML (66–76)
Peptide F	MazE	EYLCDSQGKEML (65–76)
Peptide G	MazF	KSLD (79–82)
Peptide H	MazF	QVKSLD (77–82)
Peptide I	MazF	KSLDWKAR (79–86)
Peptide J	MazF	QVKSLDWKAR (77–86)

## Data Availability

Data are contained within the article.
